# Heavy-chain antibody targeting of CD38 NAD^+^ hydrolase ectoenzyme to prevent fibrosis in multiple organs

**DOI:** 10.1038/s41598-023-49450-1

**Published:** 2023-12-12

**Authors:** Bo Shi, Asif Amin, Pranjali Dalvi, Wenxia Wang, Nicholas Lukacs, Li Kai, Paul Cheresh, Thais R. Peclat, Claudia C. Chini, Eduardo N. Chini, Wim van Schooten, John Varga

**Affiliations:** 1 Northwestern Scleroderma Program, Department of Medicine, Northwestern University Feinberg School of Medicine, Chicago, IL 60611 USA; 2 Department of Internal Medicine, The University of Michigan, Ann Arbor, MI 48109 USA; 3 Teneobio Inc., Menlo Park, CA 94025 USA; 4 Department of Pathology, The University of Michigan, Ann Arbor, MI 48109 USA; 5 Division of Pulmonary and Critical Care, Northwestern University Feinberg School of Medicine, Chicago, IL 60611 USA; 6 Department of Anesthesiology and Kogod Center on Aging, Mayo Clinic, Jacksonville, FL USA; 7 Michigan Scleroderma Program, The University of Michigan, Ann Arbor, MI 48104 USA

**Keywords:** Immunology, Medical research, Molecular medicine, Rheumatology, Rheumatic diseases, Connective tissue diseases

## Abstract

The functionally pleiotropic ectoenzyme CD38 is a glycohydrolase widely expressed on immune and non-hematopoietic cells. By converting NAD^+^ to ADP-ribose and nicotinamide, CD38 governs organismal NAD^+^ homeostasis and the activity of NAD^+^-dependent cellular enzymes. CD38 has emerged as a major driver of age-related NAD^+^ decline underlying adverse metabolic states, frailty and reduced health span. CD38 is upregulated in systemic sclerosis (SSc), a chronic disease characterized by fibrosis in multiple organs. We sought to test the hypothesis that inhibition of the CD38 ecto-enzymatic activity using a heavy-chain monoclonal antibody Ab68 will, via augmenting organismal NAD^+^, prevent fibrosis in a mouse model of SSc characterized by NAD^+^ depletion. Here we show that treatment of mice with a non-cytotoxic heavy-chain antibody that selectively inhibits CD38 ectoenzyme resulted in NAD^+^ boosting that was associated with significant protection from fibrosis in multiple organs. These findings suggest that targeted inhibition of CD38 ecto-enzymatic activity could be a potential pharmacological approach for SSc fibrosis treatment.

## Introduction

Systemic sclerosis (SSc) is a poorly-understood chronic disease characterized by synchronous fibrosis in the skin, lung and other internal organs. Fibrosis has potentially lethal consequences, and lacks effective treatment^1–7^. Persistent accumulation of activated myofibroblasts within lesional tissue, the hallmark of SSc and other forms of fibrosis, is induced by transforming growth factor-ß (TGF-ß), interleukin-6 (IL-6), and a host of other soluble factors^3,8^. The defining features of SSc, particularly skin and lung fibrosis, are notably replicated in mice treated chronically with subcutaneous bleomycin.

**Figure 1:**
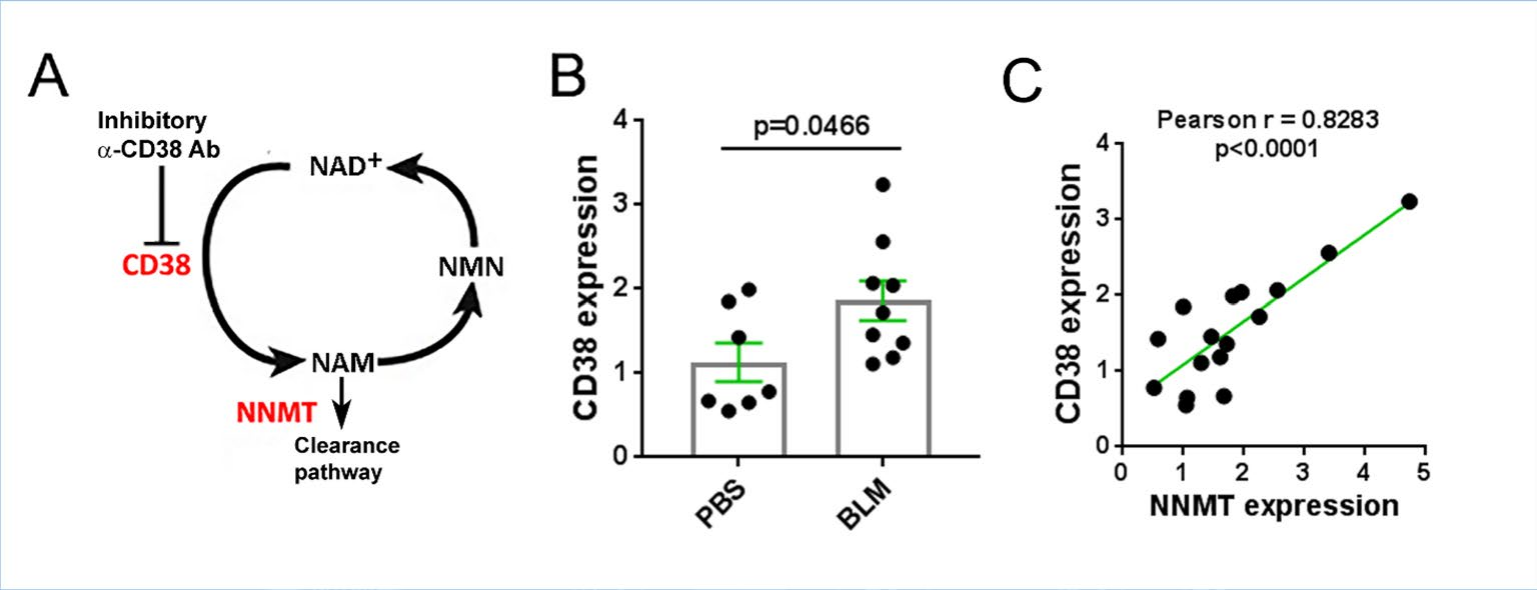
Elevated expression of NAD^+^-consuming enzymes in fibrotic skin. (**A**) Schematic diagram of NAD^+^ salvage. Red color denotes NAD^+^ consumer enzymes. (**B**, **C**) Mice were treated with daily subcutaneous injections of bleomycin (BLM) or PBS, and skin was harvested at day 21. CD38 mRNA and NNMT levels in the fibrotic skin were determined by qPCR. Each dot represents result from a single mouse, bar graph is presented as means ± SEM. (**C**) Correlation (Pearson) of expression of CD38 and NNMT.

Nicotinamide adenine dinucleotide (NAD^+^) is a critical cofactor and substrate for enzymes essential in cell signaling, damage repair, and regulation of lifespan^9,10^. A series of recent studies demonstrated that levels of NAD^+^ decline during natural aging as well as in progeroid syndromes, and decreased NAD^+^ contributes to age-dependent loss of resilience and metabolic collapse^11–16^. The widely-expressed transmembrane glycoprotein CD38 has both receptor and enzymatic functions, and serves as a link between inflammation and metabolism. In particular, we previously showed that CD38 is the main NAD^+^-hydrolyzing enzyme in mammalian tissues, and its upregulation largely accounts for age-dependent decline in NAD^+^^11,17–19^. In addition to NAD^+^, CD38 also catabolizes nicotinamide mononucleotide (NMN) and other extracellular NAD^+^ precursors prior to their intracellular transport for NAD^+^ biosynthesis (Fig. 1A)^20^. The expression of CD38 is elevated in multiple tissues during aging, as well as in various inflammatory, autoimmune, degenerative, infectious and ischemic conditions and cancer^21^. In each of these states, CD38 upregulation is accompanied by depletion of NAD^+^, which leads to impaired function of important NAD^+^-dependent enzymes resulting in cellular metabolic collapse^22^. A recent study revealed an increase in circulating CD38^+^ lymphocytes and the proportion of circulating CD38^+^ plasmablasts and plasma cells in SSc^23^. We recently demonstrated an association between elevated CD38 expression and tissue fibrosis in SSc, as well as in a bleomycin-induced mouse model of fibrosis, accompanied by a reduction in NAD^+^ in circulation and liver tissue^24^. We thus hypothesized that CD38 upregulation might have a fundamental pathogenic role in SSc fibrosis, and further, restoring NAD^+^ homeostasis will prevent the process.

The objective of this study was to investigate if selectively inhibiting CD38 NADase with a novel heavy-chain antibody can mitigate fibrotic responses in mice.

## Results

### Inhibition of CD38 NADase by Ab68 treatment ameliorates skin and lung fibrosis

**Figure 2:**
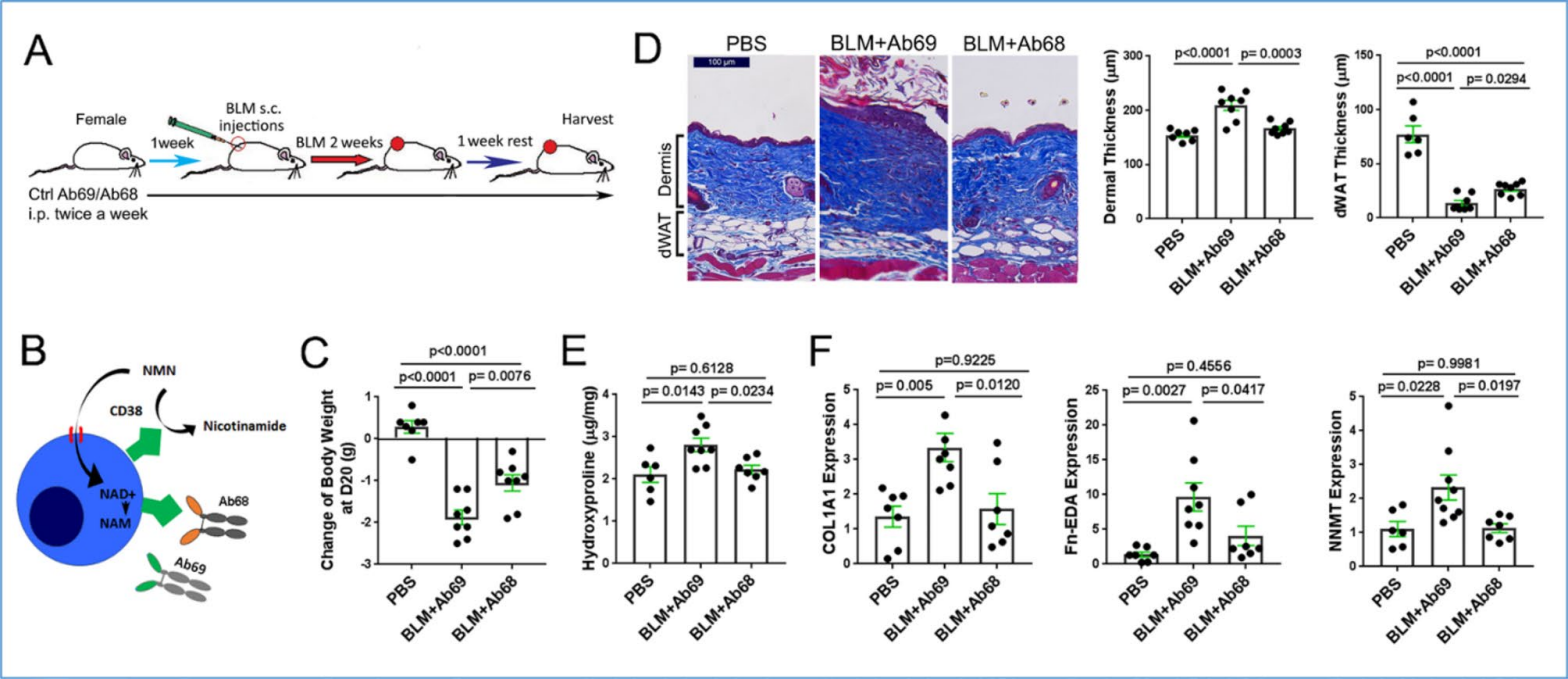
Inhibition of CD38 NADase with anti-CD38 antibody ameliorated skin fibrosis. (**A**) Schematic of experimental design. C57/BL6 mice were pretreated with i.p. Ab68 or Ab69, followed by a 14-day course of daily bleomycin (BLM) subcutaneous injections alone, or combined with Ab68 or Ab69 (7–8 mice/group). Mice were sacrificed at day 21. (**B**) Schematic of Ab68 targeting CD38 NADase. Ab68 and Ab69 are heavy-chain antibodies with human VH domains and a silenced mouse IgG2a. Both antibodies bind mouse CD38, but only Ab68 inhibits the NAD^+^ hydrolase function of CD38. (**C**) Body weight. Each dot represents the result from a single mouse. (**D**) Lesional skin, trichrome stain, representative images, left panels (bar scale = 100 μm). Quantitation of dermis thickness and dermal white adipose tissue (dWAT). (**E**) Skin collagen content. (**F**) Gene expression in skin measured by qPCR. Results are means ± SEM from an experiment representative of two independent experiments; each dot represents the mean of triplicate determinations from each mouse.

Homeostasis of NAD^+^ is governed by the action of the CD38 ecto-enzyme together with NNMT, which catalyzes the methylation of nicotinamide (NAM) generated via CD38 (Fig. 1A). In parallels with biological aging, SSc is characterized by CD38 upregulation in lesional tissues^11,24^. Bleomycin-induced skin fibrosis in mice was similarly accompanied by upregulation of CD38 (Fig. 1B). Moreover, the levels of CD38 in fibrotic skin in mice were correlated with levels of NNMT (Fig. 1C), the methyltransferase that was also significantly elevated in SSc skin biopsies^24^. We next sought to determine if inhibiting CD38-mediated NAD^+^ consumption, using the heavy-chain antibody Ab68 that selectively targets NADase ectoenzyme activity (Fig. 2B)^25^, will mitigate bleomycin-induced fibrosis (Fig. 2A). Significant weight loss (30% at day 21) seen with chronic bleomycin treatment was substantially attenuated in mice receiving Ab68 (Fig. 2C, Supplemental Fig. 1). Notably, antibody-mediated CD38 inhibition was accompanied by significant amelioration of skin fibrosis in bleomycin-treated mice. Specifically, compared to mice treated with the control antibody (Ab69), mice with Ab68 treatment showed a significant decrease in dermis thickness (Ab68 group 166 μm ± 3.8 vs. Ab69 group 209 μm ± 9.4, *p* = 0.0003, Fig. 2D), skin collagen content (Ab68 group 2.2 ± 0.1 mg/mg vs. Ab69 group 2.8 ± 0.2, *p* = 0.023, Fig. 2E) and expression of pro-fibrotic genes (Fig. 2F). Moreover, treatment with Ab68 reduced the expression of NNMT (Fig. 2F). Importantly, attenuation of dermal white adipose tissue (dWAT), a sensitive and reproducible marker of bleomycin-induced skin fibrosis, was also substantially mitigated in mice treated with Ab68 (Fig. 2D).

**Figure 3:**
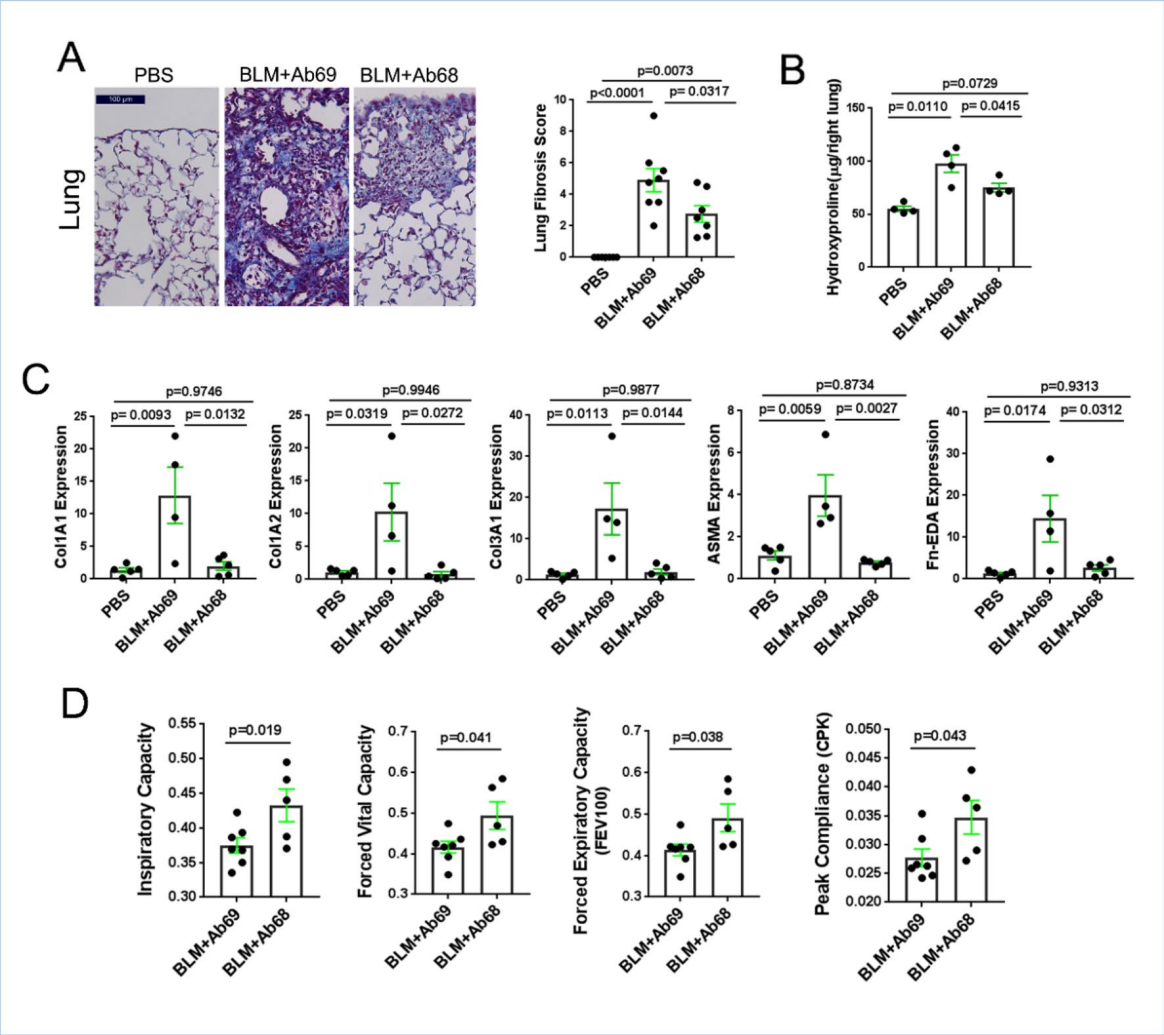
Anti-CD38 antibody treatment ameliorated lung fibrosis. For experimental design, see Fig. 2. Mice were sacrificed at day 21 and lungs harvested for analysis. (**A**) Left panels, Trichrome stain (representative images); right panel, Ashcroft fibrosis score; each dot is a single mouse; means ± SEM. (**B**) Lung collagen content (4–5 lungs/group). (**C**) Gene expression in lungs measured by qPCR; results are means ± SEM of triplicate determinations from each mouse. (**D**) Pulmonary functions were evaluated at day 21 of BLM injections. Results are shown as means ± SEM of triplicate determinations for 5–7 mice/group. Each dot represents individual mouse and P-values were determined by an unpaired Student’s *t* test or one-way ANOVA and Tukay’s post hoc test.

Chronic subcutaneous bleomycin administration was associated with adverse remodeling in the lungs (Fig. 3A). Treatment of mice with Ab68 attenuated lung fibrosis. In particular, we noted significantly lower histological fibrosis scores, coupled with reduced collagen accumulation and expression of fibrotic genes and myofibroblast markers in the lung (Fig. 3B,C). Because previous studies have demonstrated that NAD^+^ depletion can induce cellular senescence, while the senescence associate secretory phenotype (SASP) in turn can induce CD38^26,27^, it was important to examine the impact of Ab68 on p21, a widely used albeit non-specific marker of cellular senescence. Inhibition of CD38 by Ab68 treatment of the mice also reduced the number of p21-positive cells in the lesional dermis (Supplemental Fig. 2). Pulmonary function testing (at day 21 of bleomycin treatment) indicated significantly improved lung function, including Inspiratory Capacity, Forced Vital Capacity, Forced Expiratory Capacity and Peak Compliance, in Ab68-treated mice (Fig. 3D).

### Anti-CD38 antibodies selectively targeting NADase ectoenzyme boost NAD^+^ and NMN levels and augment SIRT activity

**Figure 4:**
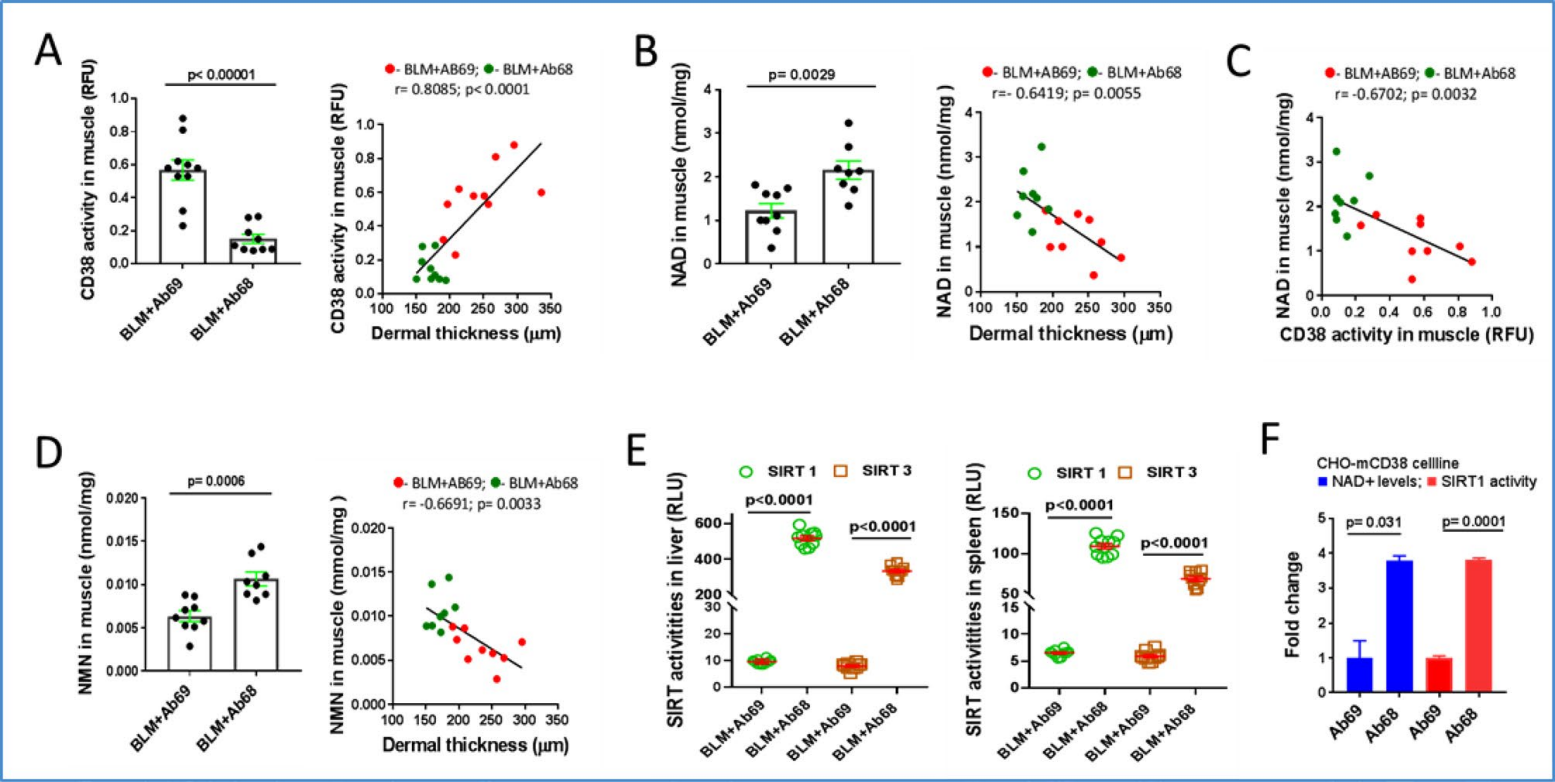
Ab68 treatment raised levels of NAD^+^ and NMN and augmented Sirt1 and Sirt3 activity. For mouse experiment design, see Fig. 2A. Mice were sacrificed at day 21 and tissue and sera were harvested for analysis. (**A**) CD38 activity in muscle and its correlation with dermal thickness. A red dot represents an individual mouse from the Ab69 group, while a green dot represents an individual mouse from the Ab68 group. (**B**) NAD^+^ concentrations in muscle, and correlation with dermis thickness. (**C**) NAD^+^ levels show negative association with CD38 activity in muscle. (**D**) NMN serum concentrations and correlation with dermal thickness. (**E**) SIRT1 and SIRT3 activities in liver and spleen. Each dot represents the mean of triplicate determinations from each mouse. The data in bar graph presented as means ± SEM. (**F**) CHO-mCD38 cells, which over-express CD38, were incubated with Ab68 or Ab69, and SIRT1/3 activity and cellular NAD levels were determined. Bars represent the means ± SEM of × 2 determinations. Pearson’s correlation is used to find a linear relationship between two variables.

We found that treatment of mice with Ab68, but not control Ab69, was associated with a decrease in CD38 activity and an increase in muscle NAD^+^ levels (Fig. 4A,B). Both tissue CD38 activity and levels of NAD^+^ were significantly associated with dermal thickness, while in muscle CD38 activity showed significant negative correlation with NAD^+^ levels (Fig. 4C). In addition, muscle NMN levels were significantly elevated in Ab68-treated mice, indicating potent systemic inhibition of CD38-mediated catabolism of NAD^+^ precursors^15^. Moreover, NMN levels in muscle were negatively correlated with dermal thickness (Fig. 4D). We have shown previously that sirtuins SIRT1 and SIRT3 have anti-fibrotic function, and their decreased tissue expression observed in skin biopsies from SSc patients, might directly contribute to the persistence of fibrosis^28,29^. Because the deacetylase activity of sirtuins is strictly NAD^+^ -dependent^30^, we examined the impact of CD38 inhibition on sirtuin function. We observed significantly increased deacetylase activity of both SIRT1 and SIRT3 in the liver and spleen of mice treated with Ab68, but not with the non-inhibitory anti-CD38 antibody Ab69 (Fig. 4E). In vitro treatment of CD38-overexpressing CHO cells (CHO-mCD38) with Ab68, but not Ab69, significantly increased both cellular NAD^+^ levels and Sirt1 and Sirt3 deacetylase activity (Fig. 4F). These findings indicate that by inhibiting CD38 ectoenzyme, Ab68 boosts organismal NAD^+^ levels, which promotes sirtuin activity, providing a potential mechanistic explanation for Ab68’s anti-fibrotic effect in mice.

## Discussion

Here we demonstrate here that selectively targeting the CD38 NADase ectoenzyme with the catalytically inhibitory heavy-chain monoclonal antibody can mitigate skin and lung fibrosis in mice. Widely expressed in both immune and stromal cell types, CD38 serves as the principal mammalian enzyme responsible for organismal depletion of NAD^+^ in ageing and diverse pathological conditions^31,32^. We recently demonstrated increased CD38 expression in skin biopsies from patients with SSc, and correlation with clinical disease severity^24^. Moreover, we found that deletion of CD38 afforded mice substantial protection from skin and lung fibrosis induced by bleomycin or from peritoneal fibrosis induced by cyclohexidine gluconate^24^. CD38 levels were markedly elevated in the lungs from patients with idiopathic pulmonary fibrosis (IPF), and genetically targeting CD38 in mice attenuated bleomycin-induced lung fibrosis^33^. Additionally, circulating CD38^+^ lymphocytes and plasmablasts were found to be elevated in SSc^23^. Our present results indicate that treatment with a heavy-chain anti-CD38 antibody selectively targeting the ectoenzyme domain, but not with an anti-CD38 antibody that does not block ectoenzyme, prevented bleomycin-induced weight loss, skin fibrosis and architectural remodeling in the lung, accompanied by reduced collagen deposition and significant improvement of pulmonary functions. We found that Ab68 treatment had effects on fibrosis and body weight comparable to those of dietary supplementation with nicotinamide ribose (NR), a NAD^+^ precursor^24^. Notably, the combination of CD38 inhibition and NAD^+^ precursor administration resulted in greater anti-fibrotic effects and protection from weight loss than either treatment alone. This is correlated with the further increase in tissue NAD^+^ levels^24^. In an environment with elevated CD38 activity, such as seen in SSc lesional skin, administering NAD^+^ precursors or enhancing NAD^+^ synthesis will have limited effect on increasing NAD^+^ levels, as elevated CD38 continues to degrade NAD^+^. In addition, CD38 degrades NAD^+^ precursors such as NMN. Inhibiting CD38 NADase activity and adding NAD^+^ precursors or increasing NAD^+^ synthesis have additive effects^11,15,25^. In the bleomycin-induced fibrosis model, the infiltration of lung CD38^+^ hematopoietic B lymphocytes, monocytes, and inflammatory macrophages as well as cutaneous F4/80^+^ macrophages was significantly increased^24^. CD38 chemical inhibitor 78c inhibited the accumulation of these inflammatory immune cells^24^. Although we do not know the exact mechanism, we hypothesize that Ab68 antibody will have the same effect-suppressing inflammation by reducing the infiltration of inflammatory cells in multiple organs.

CD38 is a 45 kDa transmembrane glycoprotein with both receptor and enzymatic functions^22,30,34^. As a membrane receptor, CD38 recognizes PECAM-1 (CD31), a constitutive marker for vascular endothelial cells. As an ectoenzyme, CD38 catalyzes conversion of NAD^+^ to nicotinamide, which is then almost completely reused for NAD^+^ synthesis via the salvage pathway (Fig. 1A). Additionally, CD38 also catalyzes the degradation of the NAD^+^ precursor NMN to nicotinamide^10,35,36^. Notably, as the main determinant governing intracellular NAD^+^ levels, CD38 regulates the activities of NAD^+^-dependent cellular enzymes^32^. Preeminent among these are the sirtuin family of deacetylases with fundamental regulatory roles in metabolism, healthspan and longevity^37^. A series of recent studies implicated sirtuins as potent negative regulators of fibroblast activation and myofibroblast differentiation, and sirtuin dysfunction has been linked to the pathogenesis of SSc and other fibro-inflammatory conditions^28,29,38–42^. In view of the role of NAD^+^ as an indispensable substrate for sirtuins and other important cellular enzymes, regulation of NAD^+^ homeostasis by CD38 has broad implications for health, longevity and aging^10^.

Expression of CD38 in immune cells is induced by lipopolysaccharides (LPS) and inflammatory cytokines such as TNF-α, IFN-γ, IL-2, IL6^43–46^. We previously demonstrated that CD38 in skin fibroblasts was upregulated by inflammatory cytokines TNF-α, IL13 as well as TGF-β^24^. The upregulated CD38 was also observed during the aging process, by secreted cytokines IL-6, interferons and soluble factors from senescent cells as part of so-called senescence-associated secretory phenotype (SASP)^21,27,36,47–50^. In fact, senescent cells do not have high expression of CD38. However, the SASP factors secreted by senescent cells induce CD38 expression and increase CD38-NADase activity in non-senescent immune cells and endothelial cells^27^. On the other hand, activation of CD38 in immune cells appeared to increase cytokine release and cellular migration toward sites of inflammation, suggesting important roles in the regulation of adaptive immune responses^51,52^. The ability of SASP secreted by senescent cells to induce CD38 NADase activity and NAD^+^ consumption in neighboring cells thus establishes a niche-specific paracrine network linking cellular senescence and NAD^+^ decline^27^. Indeed, the expression of CD38 is elevated in multiple tissues and cell types during aging, which is largely attributable to its induction by SASP^11,27^. Additionally, CD38 is upregulated in the heart following ischemia–reperfusion injury, the CNS in a variety of neurodegenerative and neuroinflammatory diseases, in tumorigenesis, and in muscle, liver and articular cartilage during aging^11,31,53,54^.

While the precise mechanism underlying CD38 upregulation in these pathological conditions and disease models are not fully understood, CD38-mediated NAD^+^ consumption leading to NAD^+^ decline and consequent loss of cellular sirtuin activity appears to be a universal mechanism underlying their pathogenesis^24,31^. We showed previously that CD38-null mice with constitutively elevated levels of NAD^+^ in multiple organs are protected from age-related metabolic dysfunction and frailty^11^. Moreover, myofibroblast activation and the development of multiple organ fibrosis are substantially attenuated in CD38-null mice with elevated NAD^+^^24^. Consistent with our results, a recent study showed elevated CD38 expression in IPF lungs, which was associated with reduced lung function^33^. Moreover, CD38 expression was elevated in alveolar epithelial cells in fibrotic lungs of bleomycin-treated young mice and increased further in old mice, which was accompanied by impairment of NAD^+^-dependent cellular and molecular activities^33^. The beneficial effects associated with reduced CD38 function can be primarily attributed to the consequent increase in NAD^+^ levels and sirtuin activity. Together these observations underscore the fundamental role of CD38-mediated NAD^+^ catabolism and subsequent NAD^+^ depletion as both key drivers as well as potential therapeutic targets in diverse aging phenotypes, organ fibrosis and other disease processes. Notably, several of the pro-inflammatory cytokines known to upregulate CD38 expression, including IL-6, interferons, TGF-ß, as well as SASP, are also implicated in SSc^2^. Thus, elevated levels or activity of these mediators might provide an explanation for CD38 upregulation noted in SSc skin biopsies^24^. Moreover, cellular senescence appears to be increased in SSc patients, and further contributes to CD38 upregulation in lesional tissues^55^. However, the precise triggers for CD38 dysregulation in SSc, their cellular origins and the underlying mechanisms, still remain to be characterized.

Ab68 may exert its anti-inflammatory effects by preventing CD38 from binding to its cognate receptor CD31, thereby reducing CD38 ^+^ immune cell adhesion to endothelium and CD31-mediated transmigration to sites of inflammation^52,56,57^. Ab68’s inhibition of CD38 ecto-enzymatic activity reduced the production of ADPR^36^, which may decrease Ca^2+^ release from intracellular depots and attenuate the chemotaxis of CD38 ^+^ immune cells^58,59^. To validate these hypotheses, further experiments are necessary.

Since extracellular NAD^+^ can be degraded into adenosine, extracellular NAD^+^ might exert some of its effects via adenosine, which can induce profibrotic effects via adenosine receptor A2a on dermal fibroblasts^60^. Depletion of adenosine has been shown to reduce fibrosis, inflammation, and vasculopathy in two preclinical mouse models of SSc^61^. The impact of Ab68 on the production of adenosine warrants further research.

Small molecule CD38 Inhibitors, derived from 4-Amino-Quinoline, are potent and non-covalent reversible inhibitors. The three primary compounds 78c, 1ai, and Iah have demonstrated specificity and potency in inhibiting CD38 and increasing intracellular NAD^+^ when administered orally^22,52^. Because CD38 is widely expressed in the brain and plays an essential role in oxytocin release^62,63^, small molecule compounds that can cross the blood–brain barrier (BBB) pose unacceptable risks of CNS toxicity and are unfit for chronic use. Ab68 antibodies (Abs) demonstrated potent NAD^+^ and NMN boosting activity^36^, are incapable of traversing the BBB, and their superior target specificity suggests a significantly safer profile than small molecule CD38 inhibitors.

In summary, the present results identify CD38 NADase as a therapeutically tractable target in SSc. Upregulation of CD38 in patients with SSc, resulting from its induction by cytokines such as TGF-ß, IL-6 and senescent cell-derived SASP enriched in the fibrotic tissue microenvironment, will promote NAD^+^ consumption and decline, which in turn impairs the function of sirtuins and other NAD^+^-dependent cellular enzymes. Reduced sirtuin activity in an NAD^+^-depleted environment contributes, both indirectly via metabolic collapse and augmented fibrotic signaling, as well as by further inducing cellular senescence, to unchecked fibroblast activation and myofibroblast persistence driving intractable fibrosis. Multiple therapeutic approaches that target CD38 in multiple myeloma, melanoma and other CD38-associated malignancies are currently investigated, with the prevailing mechanism attributed to antibody-mediated cytolysis and phagocytosis^21^. In contrast, TNB-738, a novel inhibitory antibody targeting human CD38 ecto-enzyme, is non-cytotoxic and could be used for indications where depletion of CD38-positive cells is not desired^25^. We now demonstrate that targeting CD38 with heavy-chain antibodies that selectively inhibit NAD glycohydrolase activity without inducing cell death or global CD38 blockade will boost tissue NAD^+^ and precursor levels and sirtuin activity, reduce cellular senescence and mitigate fibrosis and inflammation in multiple organs. Antibody-mediated selective targeting of CD38 thus represents a promising therapeutic strategy for SSc and related fibroinflammatory conditions currently lacking effective treatment.

## Materials and methods

### Anti-mouse CD38 antibodies (Ab68 and Ab69)

Ab68 and Ab69 antibodies UniAb clone ID337468 (Ab68) and UniAb clone ID337469 (Ab69) expressed on a silenced mouse IgG2a background are from Teneobio Inc (Newark, CA), that have been silenced to assure long half-lives, reduced immunogenicity, absence of immune effector functions, and without cytotoxic effect on CD38 positive cells. Ab68 is an inhibitor of murine CD38 NADase activity^36^. Ab69, which does not inhibit CD38 enzymatic activity, was used as a negative control^36^.

### Bleomycin-induced mouse fibrosis model and administration of anti-CD38 antibodies

All animal studies were conducted in accordance with NIH guidelines for the care and use of laboratory animals and animal protocols were institutionally approved by the Animal Care and Use Committees of Northwestern University and the University of Michigan. This study is reported in accordance with ARRIVE guidelines. Fourteen-week-old C57BL/6 female mice were provided by the Jackson Laboratory (Bar Harbor, ME). Mice were administered daily subcutaneous (s.c.) injections of bleomycin (Teva Pharmaceuticals, North Wales, PA; 10 mg/kg) or PBS for 14 days. Ab68 and Ab69 were administered intraperitoneally (i.p.) twice a week at a dose of 5 mg/kg. Mice were randomly divided into three groups (7–8 mice), as shown in Fig. 2A. Mice were weighed three time per week, and sacrificed on day 21, when lesional skin, lungs, liver, spleen, and muscle were harvested.

### Measurement of fibrosis

The amount of collagen in skin and lungs was determined by the content of the amino acid hydroxyproline using Hydroxyproline Colorimetric Assay Kits (Biovision Inc. Milpitas, CA)^64^. Paraffin-embedded mouse skin and lung samples were sectioned and stained with hematoxylin and eosin or Masson's trichrome. Dermal thickness, defined as the distance from the epidermal–dermal junction to the dermal–adipose junction or to the loose connective tissue subjacent to the panniculus carnosus, respectively, were determined at five randomly selected sites per h.p.f., as previously described^65^. Ashcroft scores, reflecting both severity and extent of lung fibrosis^66^ were determined in a blinded manner by a pulmonary pathologist.

### Measurement of pulmonary function test (PFT)

Pulmonary function testing (PFT) were performed in anesthetized mice after the insertion of a tracheal tube for mechanical breathing as previously described^67^. Buxco system was used to measure Inspiratory Capacity, Forced Vital Capacity, Forced expiratory Capacity, and Peak Compliance in tracheotomized mice. Pulmonary functions were measured at baseline for changes in lung. Statistical analyses were performed using unpaired Student’s *t* test.

### RNA isolation and qPCR analysis

Total RNA from skin and lungs was isolated by RNeasy Fibrous tissue mini kits (Qiagen, Germantown, MD, 74,704). Reverse transcription of RNA to cDNA was performed using Supermix (cDNA Synthesis Supermix; Quanta Biosciences, Beverly, MA) as described^65^. Amplification products (50 ng) were amplified using SYBR Green PCR Master Mix or TagMan gene expression assay (Applied Biosytems, Foster city, CA) on an Applied Biosystems 7500 Prism Sequence Detection System. The sequence of primer pairs used for RT-PCR were listed as below: mCol1A1 forward 5′-AGCCGCAAAGAGTCTACATG-3′, reverse 5′-CTTAGGCCATTGTGTATGCAG-3′; mCol1A2 forward 5′-CCGTGCTTCTCAGAACATCA-3′, reverse 5′-CTTGCCCCATTCATTTGTCT-3′; mCol3A1 forward 5′-CTGTAACATGGAAACTGGGGAAA-3′, reverse 5′-CCATAGCTGAACTGAAAACCACC-3′; mASMA forward 5′-ATGCAGAAGGAGATCACAGC-3′, reverse 5′-GTATTCCTGTTTGCTGATCCAC-3′; mFn-EDA forward 5′-AGTCAGTGTGGTTGCCTTG-3′, reverse 5′-CTGAACACTGGGTGCTATCC-3′; and mGAPDH forward 5′-ATCTTCTTGTGCAGTGCCAGC-3′, reverse 5′-GTTGATGGCAACAATCTCCAC-3′. TaqMan Assays gene expression for mouse NNMT (Mm00447994_m1) and for mouse GAPDH (Mm99999915_g1). Gene expression was normalized to internal GAPDH, and -fold change was calculated^68^.

### Measurement of CD38 hydrolase activity

CD38 hydrolase activity was measured in protein lysates prepared as described in Tarrago et al.^15^. Tissues were homogenized and lysed in NETN buffer (20 mM Tris-HCl (pH 8.0), 100 mM NaCl, 1 mM EDTA and 0.5% Nonidet P-40) supplemented with 50 mM β-glycerophosphate, 5 mM NaF and a protease inhibitor cocktail (Roche). After 30 min of incubation at 4 °C, the samples were centrifuged at 12,000 r.p.m. for 10 min at 4 °C. Protein concentration was determined in supernantants using Bio-rad protein assay. CD38 activity was measured using 40 μg of protein lysate and 50 μM ε-NAD as a substrate in 0.25 M sucrose and 40 mM Tris-HCl (pH 7.4). Chemical reagents were purchased from Sigma-Aldrich. Fluorescence was measured at an excitation wavelength of 300 nm and an emission wavelength of 410 nm.

### Measurement of NAD^+^ levels by cycling assay

Detection of NAD^+^ was performed as described before using a cycling assay^15,36^. To determine intracellular NAD^+^ levels approximately 15 mg of tissue was homogenized in 10% trichloroacetic acid (TCA). Samples were centrifuged at 12,000 r.p.m. for 5 min at 4 °C. The supernatants were collected, and the pellets were resuspended in 0.2 N NaOH for protein determination. TCA was removed with organic solvents (three volumes of 1,1,2-trichloro-1,2,2-trifluroethane: one volume of trioctylamine) in a ratio of two volumes of organic solvent to one volume of sample. After phase separation, the top aqueous layer containing NAD^+^ was recovered and the pH was corrected by addition of 1 M Tris (pH 8.0). For cycling assays, samples were diluted in 100 mM sodium phosphate buffer (pH 8) in a volume of 100 μl per well and added to white 96-well plates. Next, 100 μl of reaction mix (0.76% ethanol, 4 μM flavin mononucleotide, 27.2 U ml^−1^ alcohol dehydrogenase (ADH), 1.8 U ml^−1^ diaphorase and 8 μM resazurin) was added to each well. Then, 96-well plates were read in a fluorescence plate reader (Molecular Devices, SpectraMax Gemini XPS) in an excitation wavelength of 544 nm and an emission wavelength of 590 nm. We have previously demonstrated that the NAD^+^ cycling assay is as sensitive and specific as the ultra-performance liquid chromatography (UPLC)-mass spectroscopy (MS) assay^15^.

### NMN measurement by HPLC–MS

NMN was measured as described in Tarrago et al.^15^. Nucleotides were extracted in the same method as indicated for NAD^+^ analysis. The HPLC was set at a flow rate of 0.25 ml min^−1^ with 99% buffer A from 0 to 3 min, a linear gradient to 99% buffer A/1% buffer B (100% methanol) from 3 to 20 min, 80% buffer A/20% buffer B from 20 to 21 min, a linear gradient to 30% buffer A/70% buffer B from 21 to 28 min at 0.35 ml min^−1^ , 99% buffer A/1% buffer B from 28 to 31 min and a linear gradient to 99% buffer A from 31 to 37 min at 0.25 ml min^−1^. Concentrations were quantified based on the peak area compared to a standard curve and normalized to protein content in the tissue sample.

### Cell culture and measurement of cellular NAD^+^

CHO cells stably transfected with a mouse CD38 plasmid (CHO-mCD38) were cultured in DMEM medium supplemented with 10% FBS (Thermo Fisher Scientific), 2 mM L-glutamine, 120 U ml^−1^ penicillin and streptomycin (Lonza, Alpharetta, GA), and 400 nM nicotinamide mononucleotide (NMN)). At confluence, CHO-mCD38 cells were co-incubated in culture medium with Ab68 or Ab69 (31.25 nM) in 96 well plates for 24 h. Levels of cellular NAD^+^ were measured using NAD/NADH quantification kits according to the manufacturer’s instructions (Sigma-Aldrich, MAK037).

### Measurement of SIRT1 and SIRT3 activities in cultured cells and in mouse tissues

Cultured CHO-mCD38 cells, and liver and spleen from treated mice were harvested and homogenized in M-PER Mammalian Protein extraction buffer on ice as described previously^25^. The lysates were centrifuged at 4 °C for 5 min, and supernatants were incubated with anti-SIRT1 (Ab110304, Abcam) or anti-SIRT3 (Ab246522, Abcam) antibodies for 4 h at 4 °C. SIRT1 or SIRT3 was co-immunoprecipitated and eluted with elution buffer (Pierce # 21004), and their activities were measured using the SIRT GLOTM assay kits (Promega # G6450), according to the manufacturer’s instructions.

### p21 positive cells detected by immunofluorescence method in mouse skin

Paraffin-embedded mouse skins were immunolabelled with antibody to p21 (rat anti-mouse from AbCam), and immunofluorescence performed as we describe previously^64^. Images were quantified for p21-positive cells in the dermis using ImageJ.

### Statistics

Data are presented as means ± SEM. Two-tailed Student’s *t* test was used for comparisons between 2 groups. If experiment involved more than three groups, 1-way ANOVA followed by Tukey analysis was used. A P-value less than 0.05 denoted the presence of statistically significant difference. The Pearson correlation for continuous variables were used to evaluate relationships between two variables. Data were analyzed using Graph Pad prism (Graph Pad Software version 7, Graph Pad Software Inc.).

### Supplementary Information

Supplementary Figures.

## Data Availability

The datasets used and/or analyzed during the current study available from the corresponding author on reasonable request.
