# Supplementary material for: Heavy-chain antibody targeting of CD38 NAD+ hydrolase ectoenzyme to prevent fibrosis in multiple organs
Source: Sci Rep. 2023 Dec 12;13:22085. doi: 10.1038/s41598-023-49450-1 (PMC10716202; doi:10.1038/s41598-023-49450-1)
Supplement: Supplementary file 1 — Supplementary Figures. [file 41598_2023_49450_MOESM1_ESM.pdf]

Supplemental Figures

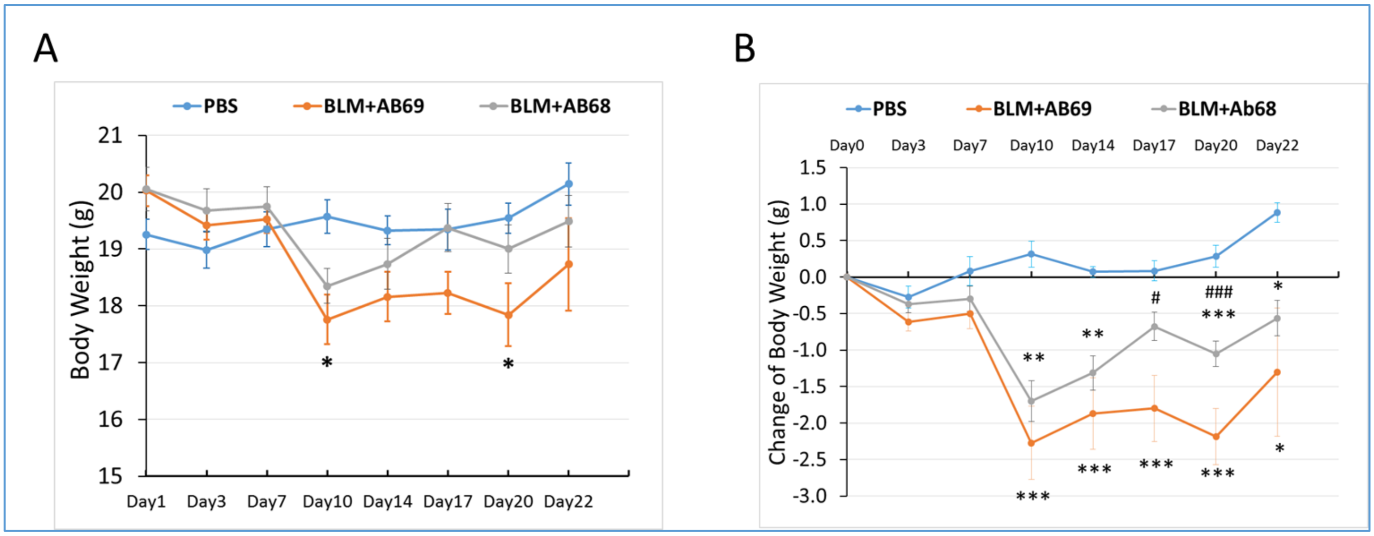

Supplemental Figure 1. Ab68 treatment attenuates BLM-induced weight loss.

Mice received daily subcutaneous administrations of bleomycin or PBS on day1 for 14 days. Ab68 and Ab69 were intraperitoneally administered twice weekly until day 21. The total body weight of the mouse **A** and the net body weight change **B** were recorded and displayed as mean ± SD. The one-way ANOVA and Tukay's post hoc test were used to determine statistically significant differences and p values. * indicates $p<0.05$, ** $p<0.01$ and *** $p<0.001$ in comparision to the PBS control. # indicates $p<0.05$ and ### $p<0.001$ when comparing the BLM+Ab68 and BLM+Ab69 groups.

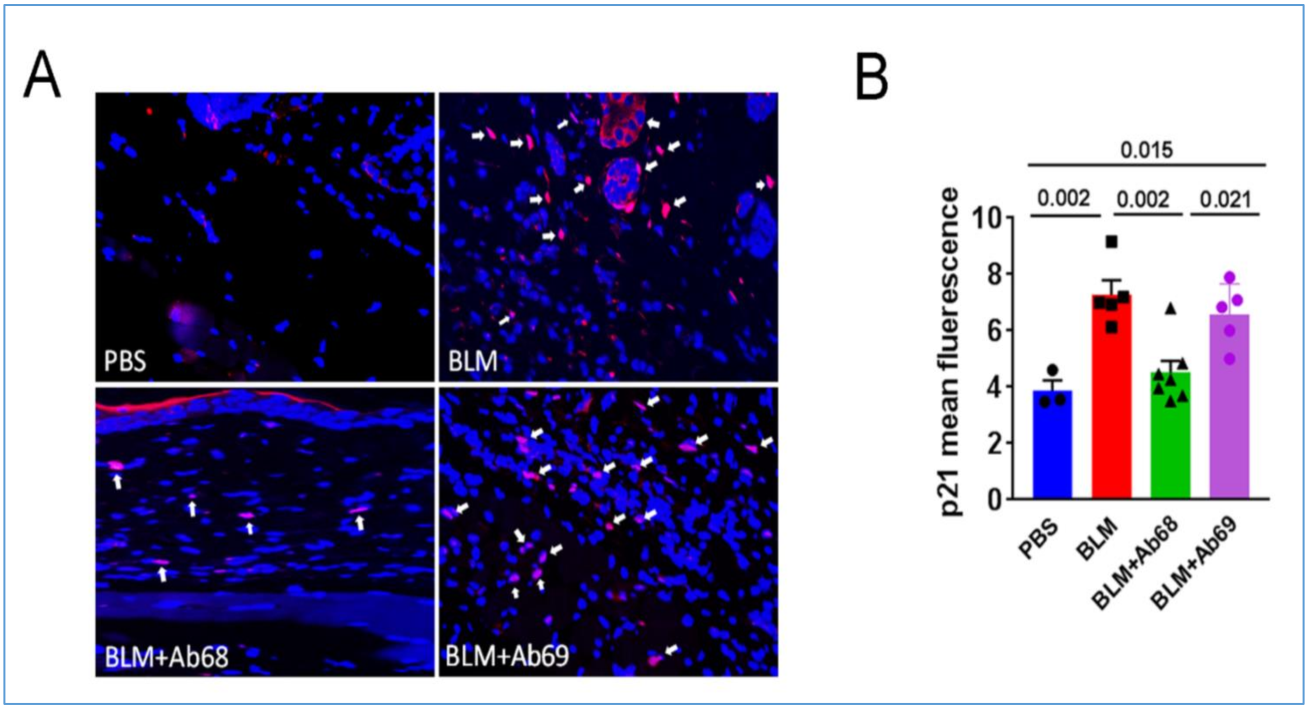

Supplemental Figure 2. Ab68 treatment reduced p21-positive cells in lesional dermis.

A. Representative immunofluorescence images of lesional dorsal skin stained with p21 antibody (Red) and DAPI (Blue). The white arrow pointed to several typical p21 positive cells. **B.** Quantification of the mean fluorescence of each group's p21-positive cells. Each dot stands for one mouse. Each bar graph displays the means ±SEM.
